# Multi-Scaled Explorations of Binding-Induced Folding of Intrinsically Disordered Protein Inhibitor IA3 to its Target Enzyme

**DOI:** 10.1371/journal.pcbi.1001118

**Published:** 2011-04-07

**Authors:** Jin Wang, Yong Wang, Xiakun Chu, Stephen J. Hagen, Wei Han, Erkang Wang

**Affiliations:** 1 State Key Laboratory of Electroanalytical Chemistry, Changchun Institute of Applied Chemistry, Chinese Academy of Sciences, Changchun, Jilin, People's Republic of China; 2 College of Physics, Jilin University, Changchun, Jilin, People's Republic of China; 3 Department of Chemistry, Physics and Applied Mathematics, State University of New York at Stony Brook, Stony Brook, New York, United States of America; 4 Department of Physics, University of Florida, Gainesville, Florida, United States of America; National Institute of Diabetes and Digestive and Kidney Diseases, National Institutes of Health, United States of America

## Abstract

Biomolecular function is realized by recognition, and increasing evidence shows that recognition is determined not only by structure but also by flexibility and dynamics. We explored a biomolecular recognition process that involves a major conformational change – protein folding. In particular, we explore the binding-induced folding of IA3, an intrinsically disordered protein that blocks the active site cleft of the yeast aspartic proteinase saccharopepsin (YPrA) by folding its own N-terminal residues into an amphipathic alpha helix. We developed a multi-scaled approach that explores the underlying mechanism by combining structure-based molecular dynamics simulations at the residue level with a stochastic path method at the atomic level. Both the free energy profile and the associated kinetic paths reveal a common scheme whereby IA3 binds to its target enzyme prior to folding itself into a helix. This theoretical result is consistent with recent time-resolved experiments. Furthermore, exploration of the detailed trajectories reveals the important roles of non-native interactions in the initial binding that occurs prior to IA3 folding. In contrast to the common view that non-native interactions contribute only to the roughness of landscapes and impede binding, the non-native interactions here facilitate binding by reducing significantly the entropic search space in the landscape. The information gained from multi-scaled simulations of the folding of this intrinsically disordered protein in the presence of its binding target may prove useful in the design of novel inhibitors of aspartic proteinases.

## Author Summary

The intrinsically disordered peptide IA3 is the endogenous inhibitor for the enzyme named yeast aspartic proteinase saccharopepsin (YPrA). In the presence of YPrA, IA3 folds itself into an amphipathic helix that blocks the active site cleft of the enzyme. We developed a multi-scaled approach to explore the underlying mechanism of this binding-induced ordering transition. Our approach combines a structure-based molecular dynamics model at the residue level with a stochastic path method at the atomic level. Our simulations suggest that IA3 inhibits YPrA through an induced-fit mechanism where the enzyme (YPrA) induces conformational change of its inhibitor (IA3). This expands the definition of an induced-fit model from its original meaning that the binding of substrate (IA3) drives conformational change in the protein (YPrA). Our result is consistent with recent kinetic experiments and provides a microscopic explanation for the underlying mechanism. We also discuss the important roles of non-native interactions and backtracking. These results enrich our understanding of the enzyme-inhibition mechanism and may have value in the design of drugs.

## Introduction

“Intrinsically Disordered Proteins” (IDPs) are proteins that are disordered either in whole or in part. They play important roles in various cellular functions, including regulation, signaling and control processes [1]. Bioinformatic and statistical studies show that many proteins are intrinsically disordered: Of the crystal structures in the Protein Data Bank that contain no missing electron density, only about 30 percent show completely ordered structures [2], [3]. From this perspective, biological function may not require ordered structure. A key question is then, how do intrinsically disordered proteins carry out biological function?

Experiment and theory are beginning to probe the relationship between the dynamics and function of highly flexible IDPs [1], [4]–[12]. The intrinsically disordered proteinase inhibitor IA3, found in the cytoplasm of *Saccharomyces cerevisiae*, is an inhibitor of the protein vacuolar yeast peptidease A (YPrA). YPrA, which is also known as saccharopepsin [13], is a member of the aspartic proteinase family. The aspartic proteinases are present in many species, including vertebrates, fungi, plants and retroviruses [14], and they play a role in a range of pathologies that includes Alzheimers disease, hypertension, malaria and AIDS [15], [16]. Until recently, few peptide inhibitors of aspartic proteinases were known [17]. Even fewer structures of inhibitor-enzyme complexes have been determined. One complex that has been studied is that of the yeast peptidase A with its naturally occurring peptide inhibitor, IA3 [18]. Free IA3 is a 68-residue peptide that lacks a stable structure in solution. Upon interaction with with YPrA, the N-terminal region of IA3 folds into an amphipathic helix that blocks the active site cleft of the enzyme. [19]–[21]. Therefore, IA3 undergoes a major disordered-to-ordered transition during binding to its target enzyme. Understanding this transition and the mechanism of IA3's interaction with YPrA may provide clues as to how IDPs regulate their function through dynamics.

Narayanan and coworkers recently used laser temperature-jump fluorescence spectroscopy and fluorescence resonance energy transfer (FRET) to investigate the kinetics of the binding-induced folding of IA3 with YPrA [22]. A rapid kinetic relaxation in IA3 was observed in the presence of YPrA, whereas this process was absent in free IA3. Modeling of the kinetic data for both free IA3 and the IA3/YPrA complex indicated that unfolded IA3 binds with YPrA prior to forming its N-terminal helix. The present work uses a multi-scaled simulation approach to explore the binding of N terminal IA3 to YPrA. (The structure of the C-terminus in the bound complex is unknown.) Although molecular dynamics (MD) simulation is a powerful tool for investigating biomolecules, the time scales for the IA3/YPrA folding and binding interaction are too long for simulation in atomic detail by MD, at least at the present time. In order to bridge the gap of time scales between experiment and computation, several approaches have been developed that reduce the number of degrees of freedom. One method is to construct a structure-based energy function at a coarse-grained residue level [23]. A second method is to identify and quantify the optimal kinetic paths between the initial disordered and final ordered native states [24], [25]. The optimal paths are those paths that connect the reactant and product on the potential energy landscape surface with the largest statistical weight [26], [27]. In this work, we first carry out a structure-based coarse-grained residue level study of IA3 binding and folding. This step uncovers the underlying thermodynamics of the binding-folding free energy landscape. We then identify several optimal paths of IA3 binding to YPrA, as initiated from different starting points, based on a fully atomistic description of the protein. We address the effect of non-native and native interactions on the binding-folding of IA3. We obtain results that are consistent with the experimental findings [22]. This multi-scaled approach provides a detailed dynamic picture of the folding of a natural peptide inhibitor in the presence of its target enzyme.

## Results

### Coarse Grained Free Energy Landscape

In order to understand the binding-folding process from a global thermodynamic perspective, we explored the free energy landscape with a coarse-grained structure-based model by MD simulation under constant temperature. In this work, the simulation temperature is chosen to be lower than the binding transition temperature so that binding is possible and the target enzyme is stable. Meanwhile a harmonic biasing potential is introduced to accelerate the sampling. The harmonic biasing potential serves two purposes: (a) It prevents the IA3 molecule from being too physically distant from YPrA. This approach prevents the molecule from consuming too much computational time wandering in free space and searching for its interaction partner. It is analogous to simulating the system in a highly crowded cell-like enviroment where IA3 has higher chances of colliding with YPrA [5]. (b) The harmonic bias also facilitates crossing of the energy barrier by elevating the free energy basin of the complex. The biasing potential enhances sampling by minimizing trapping in less probable states. This idea is similar to the conformational flooding algorithm [28]. Finally, we can find the unbiased thermodynamic properties from our simulations by transforming back from the biased to the unbiased case, using Equation 2 of the *Methods*.

**Figure 1:**
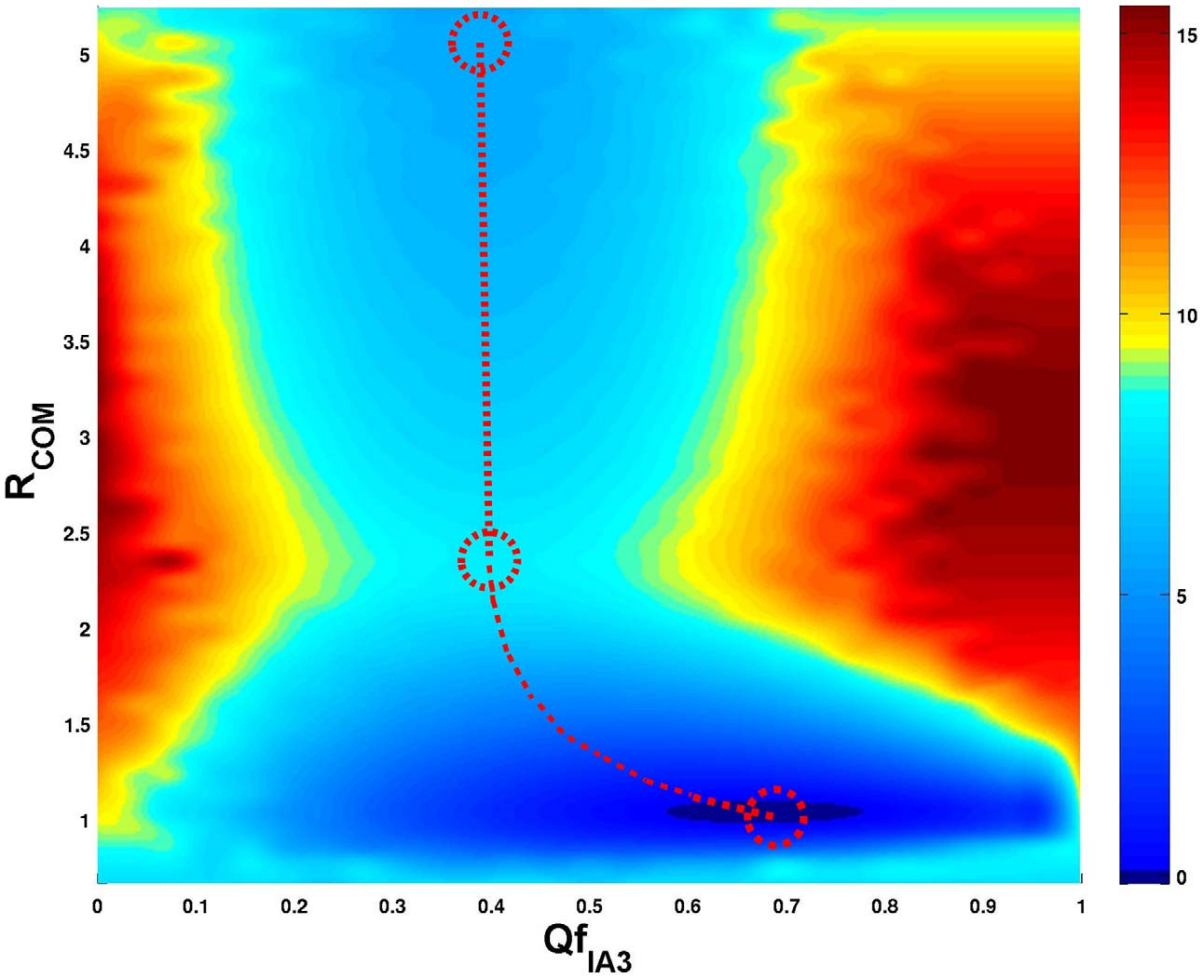
Unbiased free energy profile in terms of the IA3 folding coordinate (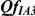) and the center of mass distance between YPrA and IA3 (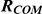 in nm), as derived from the structure-based model at the residue level.

We take the normalized native contact fraction 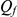 for folding of IA3 and the center of mass (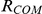) distance between IA3 and YPrA as the order parameters that quantify the progress of the folding and binding process towards the final conformation of the YPrA-IA3 complex. The free energy profile shown in Figure 1 suggests there are two stable configurations: one is the unfolded and unbound state of IA3 and the other is the native binding-folding complex. The transition state ensemble corresponds to the region where 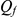 of IA3 is in the range [0.3–0.5] and 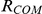 is in the range [2.2–2.5] nm. The finding that the unbound state corresponds to a nonzero 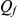, so that IA3 is not entirely disordered in unbound state, is consistent with NMR and CD measurements [17], [21], [22], which indicate that the N-terminus of IA3 is approximately 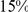 folded when the peptide is free in solution. The fact that our result for the 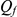 of the unbound state is larger than this value may reflect the fact that our chosen order parameter for the native contact fraction is not very sensitive to the fluctuations in the local contacts within the helical structure of IA3. We also measured the RMSD between the unbound and helical states of IA3. The average RMSD of 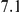 *Å* (from 31 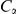 atoms) reflects the unstructured character of IA3 in unbound state. Overall, the coarse grained simulation reproduced the experimental properties of the system in a qualitative or semi-quantitative way. The free energy surface in Figure 1 indicates that binding and folding of IA3 are decoupled, with no folding occuring as the system approaches the transition state region. After the transition state however the binding and folding become strongly coupled. IA3 first approaches YPrA through binding from distant initial positions, then overcomes the transition state barrier, and finally folds itself into the structured conformation. Binding precedes folding.

### Transition State and Key Residues Analysis

From the free energy profile in Figure 1 we can conclude that IA3 binds prior to folding. Here we address the question of which regions of YPrA interact with IA3 at the transition state.

**Figure 2:**
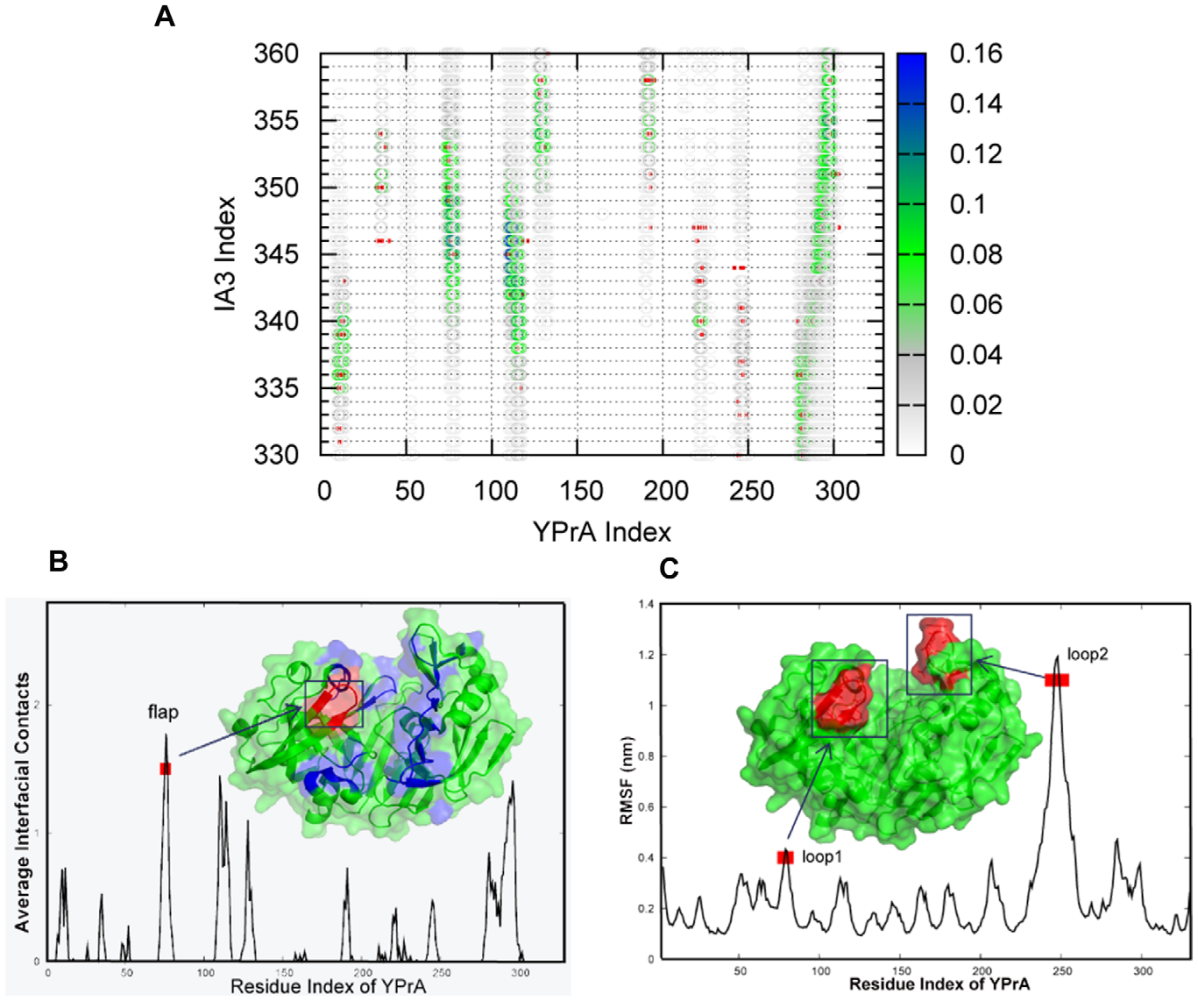
Transition state analysis obtained from coarse-grained structure-based model. (A) Binding contact map based on cut-off algorithm instead of 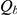. Native contacts are indicated by red squares. (B) Distribution of the average number of interfacial contacts. Contacts are primarily formed at the surface of active site groove of YPrA. The contacting residues are colored in blue in the cartoon. (C) RMS fluctuation of the 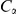 atoms as a function of residue number, as found by MD simulation. This provides a measure of the flexibility of local regions of YPrA.

We captured the contacts between IA3 and YPrA by using the cutoff algorithm instead of counting only the native contacts 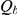. Figure 2A shows that the interfacial contacts at the transition state are distributed widely with low populations. Many of these contacts do not coincide with the native contacts (labelled by red square points) in the PDB structure of the IA3/YPrA complex. This implies that the transition state may be characterized by many non-native contacts and only a few native contacts. The important role of non-native interactions in the early stages of IA3 binding to YPrA can not be captured quantitatively by the structure based residue-level model, but it is explored in our full-atomic model, which uses a physics-based force field whose energy function combines the AMBER and OPLS force fields. Figure 2B shows the distribution of interfacial contacts in the transition state. Contacts are mostly formed at the surface of active site groove of YPrA, which is shown in blue in the cartoon representation. This distribution shows unambiguously that the first stage of the interaction involves IA3 binding to the surface of the active site groove. The highest peak, colored in red for emphasis, corresponds to the “flap” region, a 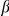 hairpin loop formed by residues 72–82, which project out to cover the YPrA active site. This structural motif is commonly found in aspartic peptidases [15].

At our simulation temperature, YPrA is not a completely rigid partner in IA3 folding and binding. Figure 2C shows the effect of temperature in the RMS fluctuation in several local regions of YPrA. X-ray experiments [29] also show that the electron density is poor at the two loop regions marked with red squares in the figure. These two loop regions are the “flap” (or loop1) and a second region, named loop2. Comparing Figure 2B with Figure 2C shows a role for the “flap” region in controling IA3 binding to YPrA. The “flap” region forms the most contacts with IA3 although the RMS fluctuation data does not indicate a large capture radius. By contrast, loop2 has a largest capture radius as reflected by its structural fluctuation during binding, but it does not contribute to the interfacial contacts with IA3. Remarkably, at the tip of the flap, there is one absolutely conserved tyrosine (Tyr75) that is considered to play a crucial role in the capture and cleavage of substrates [30].

### What Happens after Binding?

**Figure 3:**
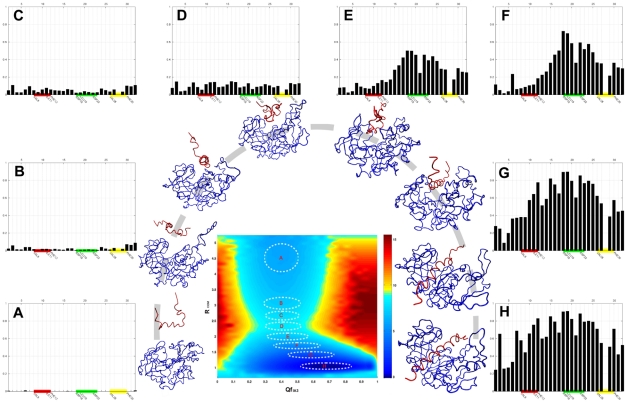
The evolution of the fraction of native interfacial contacts of N-terminal IA3 along the binding routes, as obtained by the coarse-grained structure-based model. Nine hydrophobic amino acid residues in IA3 (V8, I11, F12, L19, A23, V25, V26, A29 and F30) form three hydrophobic clusters. These are “cluster-1” (red), consisting of V8- X-X-I11-F12 in the N-terminus; “cluster-2” (green), consisting of L19-X-X-X-A23 in the mid region, and the C-terminal “cluster-3” (yellow), consisting of V26-X-X-A29-F20. These hydrophobic clusters are indicated by color along the abscissa. The plots showing evolution of 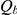 are placed around the two-dimensional free energy landscape, from unbound state to the complex state, and are labelled A to H correspondingly. Typical conformations at those locations on the landscape are also shown.

To gain further insight into the process that follows IA3 binding with the surface of active site groove, we investigated the distribution of the native interfacial contact fraction of individual IA3 residues (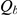) along the binding routes. In the crystal structure of the complex, the hydrophilic face of IA3 is oriented toward the solvent. The other face of IA3 is composed of the nine hydrophobic amino acid residues, V8, I11, F12, L19, A23, V25, V26, A29 and F30. This face is enveloped completely with the residues of the YPrA active site cleft and consists of three hydrophobic clusters: “cluster-1” (red) of V8-X-X-I11-F12 in the N-terminal, “cluster-2” (green) of L19-X-X-X-A23 in the mid region, and the C-terminal “cluster-3” (yellow) of V26-X-X-A29-F20 (see Figure 5 in Text S1). These clusters are indicated in Figure 3, which shows the evolution of the native interfacial contact fraction (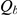) of individual IA3 residues. We find that 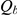 is well-distributed and less than 0.2 at the transition state region. By following the evolution of distribution along the binding routes we see that the mid region of IA3 forms native contacts with YPrA first, followed by the C-terminal region, and finally the N-terminus. However, the distribution of IA3 intrachain contacts does not show a sequential order of IA3 folding. It seems that the folding of IA3 does not necessarily occur from a particular nucleation site.

### Binding-Folding Path Revealed in Atomic Details

In studying protein folding and binding, the Q score (defined in the Text S1) for structural similarity has been extensively used as a structural reaction coordinate [31]–[34]. Q represents the fraction of native contacts that have been formed and it characterizes the structure's similarity to a referenced structure. Here, the referenced structure is the crystal structure of the enzyme-inhibitor complex of YPrA-IA3 (PDB code: 1DP5). To monitor the folding and binding of IA3 interacting with YPrA in a fully atomistic description, optimal kinetic paths were calculated in order to determine the most probable pathways between the beginning and ending points. The optimal paths depend on the choice of initial and end points. For the end point we use the structure of the native IA3-YPrA complex, as resolved by xray crystallography. The initial point is disordered, unfolded IA3 and uninhibited, folded YPrA. Obviously, the initial point for IA3 should consist of an ensemble of conformations with a sufficient number of degrees of freedom. Unfolded conformations of IA3 were generated by molecular dynamics with explicit solvent at high temperature. Three paths were chosen to illustrate the folding and binding process in detail. We refer to them as 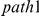, 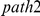 and 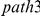.

**Figure 4:**
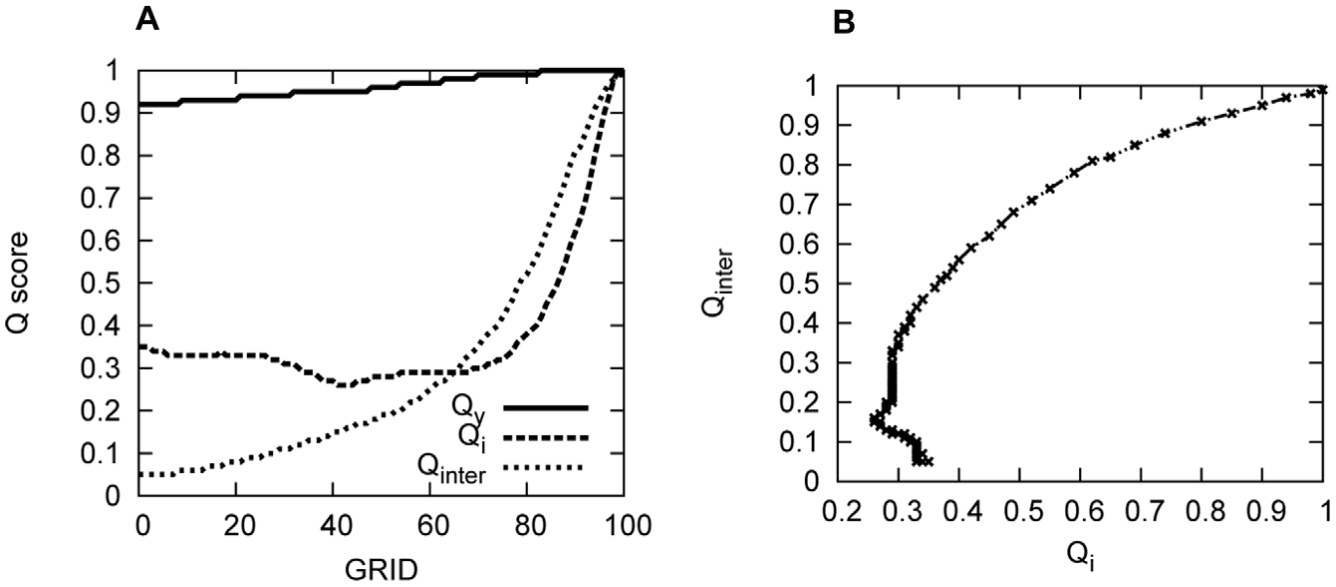
Folding and binding scores in 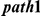, as obtained by the full atomic model. “GRID” refers to the discrete conformations along the kinetic pathway. The evolution of Q scores is shown in (A). The relationship between the IA3 folding score 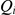 and the IA3/YPrA binding score 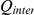 is plotted in (B).

Figure 4 shows 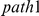. 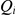 and 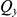 are the intrachain contact Q scores of IA3 and YPrA, respectively. The Q score of the interaction between them (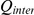) represents the interfacial similarity relative to native binding complex of IA3 and YPrA. Figure 4A shows that the 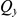 curve increases slowly close to 1, corresponding to the native inhibited structure. YPrA does not move much although it manifests some flexibility to accomodate the folding of IA3, mostly in the two loop regions located on the surface of the active site groove. Figure 4B shows the evolution of the folding score 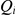 and binding score 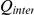 along the path. 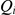 does not vary much when 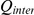 is less than 0.35. It even decreases slightly (IA3 unfolds) before grid 70 (where folding begins) due to backbone movements, not helix formation and breaking. This is consistent with the experimental indications that the pre-equilibrium of folding may not be helpful to IA3 folding through binding [22]. 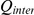 increases more and more along the path, especially when it exceeds the 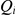 of IA3 after grid 64 and reaches 0.5 at grid 80. It implies that IA3 does not fold itself before binding tightly with YPrA. The binding score of IA3 to YPrA as a function of the folding score 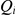 is shown in Figure 4B. Before entering into the hydrophobic cave of YPrA, IA3 searches the structural surface of YPrA with a continuous adjustment of its positioning, as reflected by the zigzag behavior of 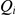, until the binding score reaches 0.5. Folding proceeds once significant binding is realized on the interface, and binding and folding are subsequently coupled as the final native complex forms. The evolution of the structures and the related contact maps are shown in Figure 4c in Text S1.

### Common Scheme Revealed by Multiple Pathways

The Q score measures only the residue-level similarity of the backbone to a reference structure. Therefore we introduce a Shadow Contact Map (SCP), an algorithm that calculates the interatomic contacts involving sidechain atoms [35]. The SCM algorithm describes the contact map excluding unphysical contacts. A cutoff distance is used to define the contacts, and this cutoff is set as 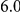 *Å* in our calculation. As in the definition of Q score, the total number of contacts can be divided into monomeric folding contacts and interfacial contacts. The contacts are grouped into two categories: native and non-native contacts, as determined by whether the contacts between residue pairs exist in final conformation. For final state, the atomic interfacial contact number and the intrachain contact number of IA3 are 604 and 103, respectively. This indicates that the interfacial interactions are far stronger than the intrachain interactions in IA3. This may explain why the kinetic process proceeds as binding followed by folding.

**Figure 5:**
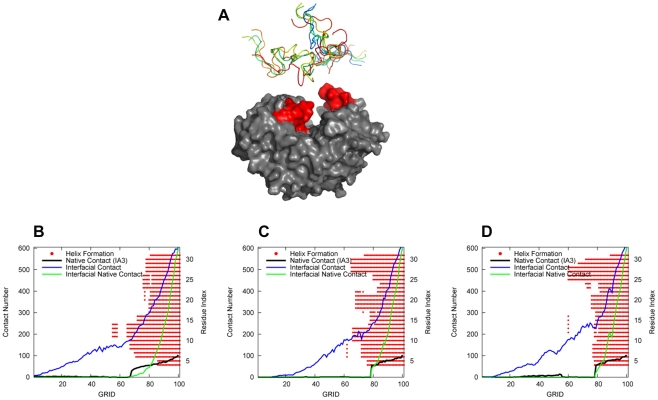
Kinetic pathways by full atomic model. (A) The initial structures for the binding pathways calculated. Different initial unfolded structures of IA3 are represented by coils with different colors. The two loop regions of YPrA are indicated in red. The evolution of atomic contact number and helix formation along the folding pathway are shown for 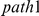 (B), 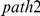 (C) and 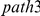 (D). In these panels, the number of native contacts of IA3 is shown by the black line. The total interfacial contacts and the native interfacial contacts are shown in blue and green lines respectively. The red bars indicate IA3 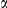 helix formation, with the residue index on the right axis. For clarity, other secondary structural elements are not shown.

The evolution of the number of atomic contacts and the helix formation in IA3 along the folding pathway of 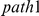, 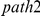 and 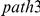 are shown in Figure 5B–5D. For exploring the relationships between atom-atom contacts and IA3 folding, the contact number curves are overlaid with the evolution of 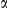 helix formation of IA3. Residues constituting 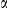 helix are assigned via analysis by the DSSP program [36], according to characteristic hydrogen-bond patterns, but other secondary structure elements such as coils and turns are excluded from this plot for the sake of clarity. Note that we have not detected beta sheet elements in our model, although there are experimental reports that IA3 may bind pepsin as a beta-strand and is therefore cut and digested as a substrate [19]. From the evolution of number of interfacial contacts (blue line) and the native contacts in IA3 (black line), we can see that IA3 binds with YPrA more and more tightly before it begins to form native contacts and helix structure. The native interfacial contacts then (green line) begin increasing until most of native contacts in IA3 are formed. For 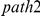 and 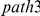, the evolution of contact number is quite similar, but the corresponding processes of helix formation are significantly different. The long helix is formed from three nuclei located around the three hydrophobic clusters. Although there are significant differences between the three pathways, they reveal the common theme that IA3 binds to YPrA prior to folding. We also see clearly that non-native interactions are the dominant driving force in the initial stage of binding. Non-native contacts smoothly increase, while the native interface and native folding contacts only begin to appear at grid values near 70.

### The Role of Non-Native Interactions Revealed by Pathway in Average

**Figure 6:**
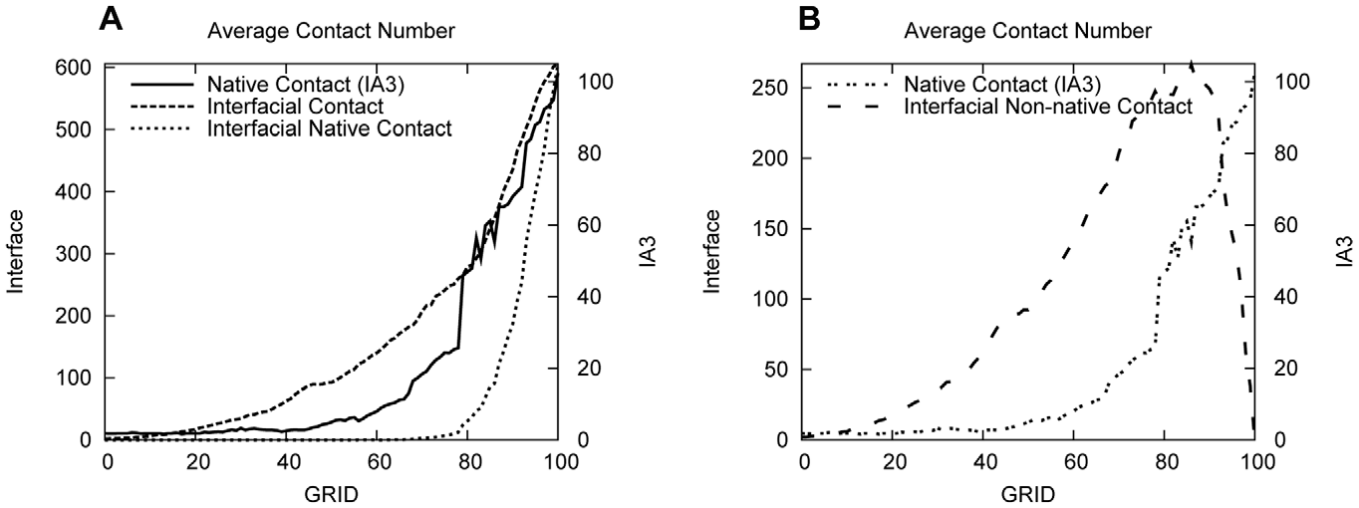
Evolution of the average number of atomic contacts along all the pathways we calculated. (A) The development of native contacts of IA3 is overlaid with curves showing the formation of interfacial contacts, both total and native. The native contacts of IA3 are shown by the black line. The total interfacial contacts and its native contacts are shown by the dashed line and dot line, respectively. (B) The development of IA3 non-native interfacial contacts, as well as native interfacial contacts, as a function of progress along the kinetic pathway.

The average path is shown by the evolution of the average number of atomic contacts in Figure 6. A sharp increase in IA3 native contacts is observed in the black curve. This can be explained as the result of contact network forming in a highly synergistic way. Figure 6A shows that the native contacts of IA3 form together with non-native interfacial contacts while the number of interfacial native contacts remains nearly zero until grid 80. At the first stage of kinetic binding, the interactions between IA3 and YPrA are mostly contributed by non-native contacts. It is the non-native interactions between IA3 and the residues on the surface of YPrA that induce IA3 to bind with its target partner. Therefore, non-specific (non-native) interactions induce initial binding of IA3 to YPrA. After IA3 reaches YPrA, native interactions of binding set in by adjusting the conformation at the active site groove.IA3 folds into helical structure after binding with the active site groove of its target enzyme.

### Why Backtracking?

It is noteworthy that in 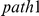 (see Figure 5B) we observe a certain fraction of the helix content formed between grid 55 and 58 disappears and then later reappears. The formation and breakup of local secondary structure is observed not only in 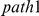, but also in several other pathways that we calculated. In addition, we also found the formation and breaking of native contacts in 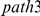 (see Figure 2c in the Text S1). Remarkably, an analogous process was observed in the investigation of the folding of Interleukin-1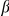 (IL-1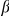) [37], [38], knotted proteins [39], CheY-like family [40], [41] and SAM-1 Riboswitch [42]. This behavior is known as “backtracking”. Here, we define it as the interim formation of local secondary structures or native contacts along the reaction pathways. Experiments in 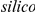 and in 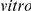 suggest that it is the result of topological frustration. Here, we propose that it results not only from topological factors but also from energetic contributions to the stability of IA3, as a partial compensation of entropy reduction during binding to YPrA.

During a biologically realistic interaction, binding to the special partner can help a protein to shrink the search space in the energy landscape. However, the associated free energy increases due to the rapid entropy reduction. As a partial compensation, occasional interactions may form only if they are energetically favored, irrespective of whether they are native contacts. These interactions do not form optimally but form easily in certain topologies. Therefore they are often unstable and fragile. During this process, both native interactions which are not stably formed and non-native interactions which are not included in the final state play roles in smoothing the free energy landscape. They stabilize the protein energetically, thus compensating the entropy reduction. As the molecule searches deeper in the free energy basin, the unstable native interactions will break down and reform stably in final structure. Hence backtracking is observed.

However we do not always observe non-native interactions or backtracking of native interactions along folding or binding pathways in nature. We explain this from three perspectives. First, the order parameters are usually coarse. They are not accurate enough to capture these details. Second, these interactions are transient and unstable. They may be difficult to measure. Third, not all the pathways are very rough. In this case of IA3-YPrA, it seems that non-native interactions play a more important role in IA3 binding while backtracking interactions are more significant in IA3 folding. From this, we believe non-native interactions and unstable native interactions can both play a role in protein folding and protein-protein recognition.

## Discussion

Like many IDPs, IA3 forms an ordered structure in the presence of its interaction partner. Its binding and folding dynamics play an essential role in the regulation of its target enzyme, YPrA. Molecular dynamics simulations can help us to explore the interaction at a level of detail that is difficult to obtain in laboratory experiments. However, standard MD is often limited by the temporal range it can probe at atomic detail. In this work, we developed a multi-scaled approach to provide a comprehensive picture of the protein binding-folding dynamics, including both global thermodynamic landscape and atomic details of structural evolution paths.

Several reaction pathways were generated from different starting points to the final conformation of the protein-inhibitor complex. Although there are significant differences between the multiple pathways, which reflect the multidimensional nature of the underlying energy landscape [23], [25], [27], [43], all reveal a common theme that IA3 binds to its target enzyme prior to folding itself into a helix. This finding is consistent with that of a coarse-grained free energy landscape from a structure-based MD simulation. In summary, the following folding and binding mechanism emerges. In the first step IA3 moves close to YPrA and binds to the surface of the active site groove via non-native interactions, through the long range electrostatic attraction. Before overcoming the free energy barrier, most of IA3 remains unstructured. Once IA3 enters into the cleft, its motion is greatly restrained, due to the lack of space for motion. In this highly hydrophobic environment, IA3 finally folds into an amphipathic 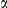 helix at the long cleft. In addition, we found that the mid region of the IA3 sequence, consisting of hydrophobic 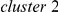, forms native interactions with YPrA earlier than the two terminal regions. This may be the result of stabilization by the interactions with the YPrA “flap”. During binding, YPrA plays the role of a template to induce IA3 folding into the characteristic structure that blocks the active site of the enzyme. In other words, the mechanism of saccharopepsin inhibition by IA3 as revealed by our simulation is in favor of the “induced-fit” model [12]. In this context, an “induced-fit” mechanism refers to a target enzyme that induces in its inhibitor a significant conformational change.

We also examined the non-native interaction by classifying the atomic contacts, as calculated by a new algorithm (SCM). At the first stage, the interactions between IA3 and YPrA are under the control of non-native interfacial contacts. The recognition process of the inhibitor-enzyme complex is dominated by these non-native interactions, which have been reported to play a role in protein assembly [9], [44]–[48]. The great success of simulating protein folding using structure-based models [49] which depend on the native topology suggests that the native contacts govern the folding of a protein that is well-designed by evolution. In the conventional view, non-native interactions are the major factor contributing roughness to the energy landscape [31], [46]. Why does non-native interaction seem to play a facilitating role for binding in the IA3/YPrA system? It is easy to explain the results in the view of the structure of the enzyme-inhibitor complex. As the target binding site is located deep in the groove, IA3 has to search the molecular surface of YPrA to find an entropically favored and energetically optimized path to the hydrophobic cleft. Experimental studies have already hinted that non-native contacts from the helix-forming (enzyme-inhibiting) N terminus as well as the disordered C terminus (not included in this study) of IA3 assist the kinetics during early stages of the interaction without affecting the final stability of the complex [17], [20], [22]. The importance of nonnative interactions was also observed in pKID and KIX binding experiments [1], [4], [9] and DNA-binding proteins [48]. These earlier findings support our conclusion here as well as the fly-casting mechanism [50].

From the energy landscape perspective, the underlying landscape of the entire binding process must be funnel-like in order to guarantee biological recognition and native binding complex formation. There are several ways to guarantee the underlying landscape to be funnel-like [23], [51]. One way is to enhance the native interactions or native bias. The other way is to reduce the non-native interactions or the roughness of the landscape. Those two ways are natural and conventionally emphasized. However there is another way to help the formation of the funneled landscape. A reduction in the entropy can significantly shrink the search space of the landscape. Here we see non-native interactions, even if not energetically favored, can contribute significantly to forming the binding funnel by reducing the entropy, bringing IA3 closer to the target YPrA interface). In this sense, non-native interactions can help the binding process.

Here we reveal the interaction mechanism of an aspartic proteinase and its endogenous inhibitor. Our studies provide a greater understanding of this unprecedented mode of enzyme inhibition. The results demonstrate the success of the multi-scaled approach for explorinng the interaction of IA3 and YPrA, and they are consistent with the conclusions from time resolved experiments, which suggest non-specific binding followed by folding [14]. The combined method may be useful in understanding other enzyme-inhibitor systems. It also may offer valuable insights into the design of drugs inhibitors for the aspartic proteinases generated by pathogenic organisms.

## Methods

### Coarse-Grained Structure-Based Model

We performed the molecular dynamics simulations using a structure-based Hamiltonian to describe the energy of the protein in a given configuration. A structure-based Hamiltonian takes into account only native interactions, and each of these interactions enters into the energy balance with the same weighting. Therefore the model does not have heterogeneity in energy and it includes only topological frustration. Each amino acid is described by a single bead on a polymer chain located on the 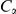 position [52]. The 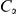 structure-based Hamiltonian is given by the expression:
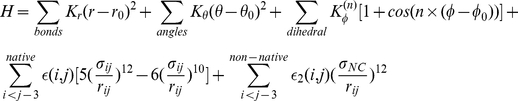



The total energy is divided into bond stretching, angle bending, torsion and nonbonded interactions. 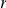, 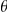 and 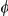 are the virtual bond length, bond angle, and torsion angle defined by 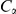 position. 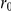, 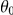 and 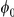 are the corresponding native values from the PDB structure. Nonbonded interactions are considered when two 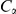 atoms i and j are separated sequentially by at least three residues on a chain or when they come from different chains, are subdivided into native interactions and nonnative interactions. For native contacts, 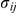 is the distance between the 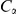 positions of contacting residues i and j. For non-native contacts, 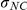 provides excluded volume repulsion. We treat the nonlocal interactions within a chain and between the chains with the same strength. The native contact map is derived from a shadow contact map (SCM) [35]. Parameters 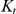, 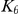, 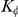, 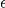, 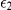 weight the relative strength of each kind of interactions contributed to energy, and 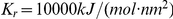, 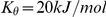, 
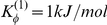
, 
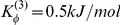
, 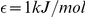, 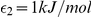. In order to sample more binding transitions we added a bias potential into the Hamiltonian. The bias potential is intended to make binding transitions more frequent by raising the free energy of the bound state. Here, we choose a harmonic form where the bias potential energy depends on the center of mass distance (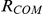) between YPrA and IA3.
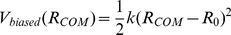
(1)Here 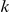 is the force constant, 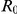 is the equilibrium position, 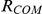 is the COM distance between the two chains. Then the Hamiltonian has a new form 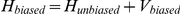. In the native complex, where IA3 is bound to YPrA, 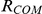 (only 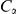) is 1.063 nm, while for 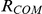 larger than 3.5 nm, we consider the system to be in the unbound state. We choose 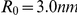 to ensure that the bias potential lifts the free energy of bound states more than unbound states. Through many trials we found that an optimized value for the force constant is 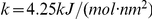.

The unbiased thermodynamic average of a function 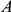 can be calculated as follows:
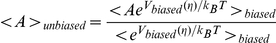
where 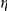 is the reaction coordinate. The free energy of the system at 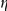 is given by 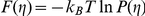, where 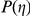 is the equilibrium probability.
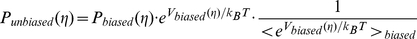
The unbiased free energy can be calculated as
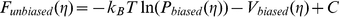
(2)The constant 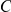 does not change in constant temperature simulations, so in our simulations we can select the value that sets the lowest 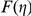 to zero.

The simulations were performed using the Gromacs software package [53]. We put the protein system in a 50 nm cubic box corresponding to a low protein concentration. In fact, the effective box length is about 8.4 nm (the largest 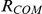 in coarse grained MD simulation). Nonbonded interactions are cut off at 3 nm. The time step was 0.5 fs. Stochastic dynamics were used with a drag coefficient 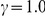. We started our trajectories with 9 different configurations in either native or nonnative state. The actual total constant temperature simulation time is 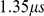. The total data include 214 binding and dissociation transitions, allowing us to observe how the dynamics change during the folding and binding. We calculated the free energy from the trajectories using WHAM (Weighted Histogram Analysis Method) [54], and using the formula 2 to get unbiased free energy. The simulation temperature is set at 176 K. Plotting the 2-D free energy surface for the binding/folding behavior requires two independent reaction coordinates, representing binding and folding respectively. From the transformation equation 2 we know that 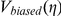 has an explicit expression only when 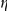 contains 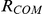. 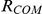 can describe the binding behavior. For folding, we choose 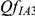, which is defined as the fraction of native spatial tertiary contacts. A native contact is formed if the distance between the two 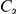 atoms is shorter than 1.2 times their native distance 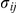.
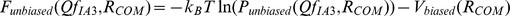



### Simulation Details of Optimal Paths Calculation at Atomic Level

The free and inhibited states of YPrA were generated from crystal structures taken from the Protein Data Bank (1FMU and 1DP5, respectively). The unfolding of IA3 was generated by Langevin dynamics by NAMD with the Charmm32 force field. Then, the initial and final structures of the complex were modelled. Crystallographic water molecules and carbohydrate moieties were removed. After modeling of the reactant and product, the paths connecting these states were calculated with the MOIL package [55]. The MOIL energy function combines the AMBER and OPLS force fields [56], [57]. We can solve the minimum energy path if the pre-specified initial and final states are known. Given the minimized endpoint structures, the initial guesses for the trajectory are determined by the minimum-energy-path self-penalty walk (SPW) [58] functional embedded in the CHMIN module. Then these paths were optimized in the SDP module with steepest descent. The solvation effects are described by the Generalized Born model [59], [60]. The high-frequency modes from the trajectories are filtered and modeled as Gaussian white noise. The cut-off distance for van der Waals interactions is 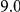 *Å*.

### Steepest Descent Path Algorithm

The steepest descent path is widely used in qualitative interpretation of chemical reactions [61], [62]. In analogy to the classical action, an action 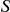 as a function of length in a discrete representation is defined to represent a most probable Brownian trajectory as follows:
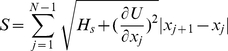
(3)


We split the path by N grids to approximate the path by a set of discrete conformations. 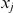 is the entire vector of conformational coordinates at grid 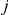. The initial conformation is 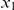 and the final conformation is 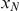. The potential energy 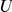 is a function of the mass-weighted coordinate vector. The constant 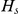 is an arbitrary positive value that mimics the energy in classical mechanics. Optimal paths with different thermal energies are generated by tuning this parameter. The steepest descent path is the limiting path that optimizes the action for 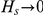. The shortest path between 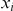 and 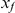 as generated by linear interpolation is the optimal path for 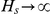. Here we used 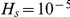.

Given the two end structures, the SDP module will minimize the target function:
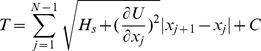
(4)

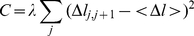
(5)

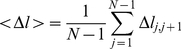
(6)where C is a restraint to ensure that configurations 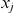
's are distributed approximately uniformly along the pathway. The target function 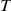 is minimized by conjugate gradient local minimization. 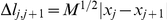 is the arc-length of the path in mass weighted coordinates between conformation 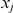 and 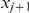. 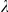 is the strength of a penalty function that restrains the step length to the average length 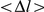. For further details see Ref. [63].

SDP is a continuous curve with a low-energy barrier that connects the reactants and the products. An important advantage of an SDP is that it allows testing of a concrete mechanism. The disadvantage is that it gives no information about the properties of the system far from the steepest descent path. Other non-native interactions which are away from the binding groove cannot be sampled on the steepest descent path. As we know, the Milestoning method [64], [65] has been developed by Ron Elber and coworkers to solve this problem. However, it is still an open question how to calculate kinetics and thermodynamics of long-time biological processes, which are typically not accessible by straightforward MD simulation.

### Boundary Structures Preparation

**Figure 7:**
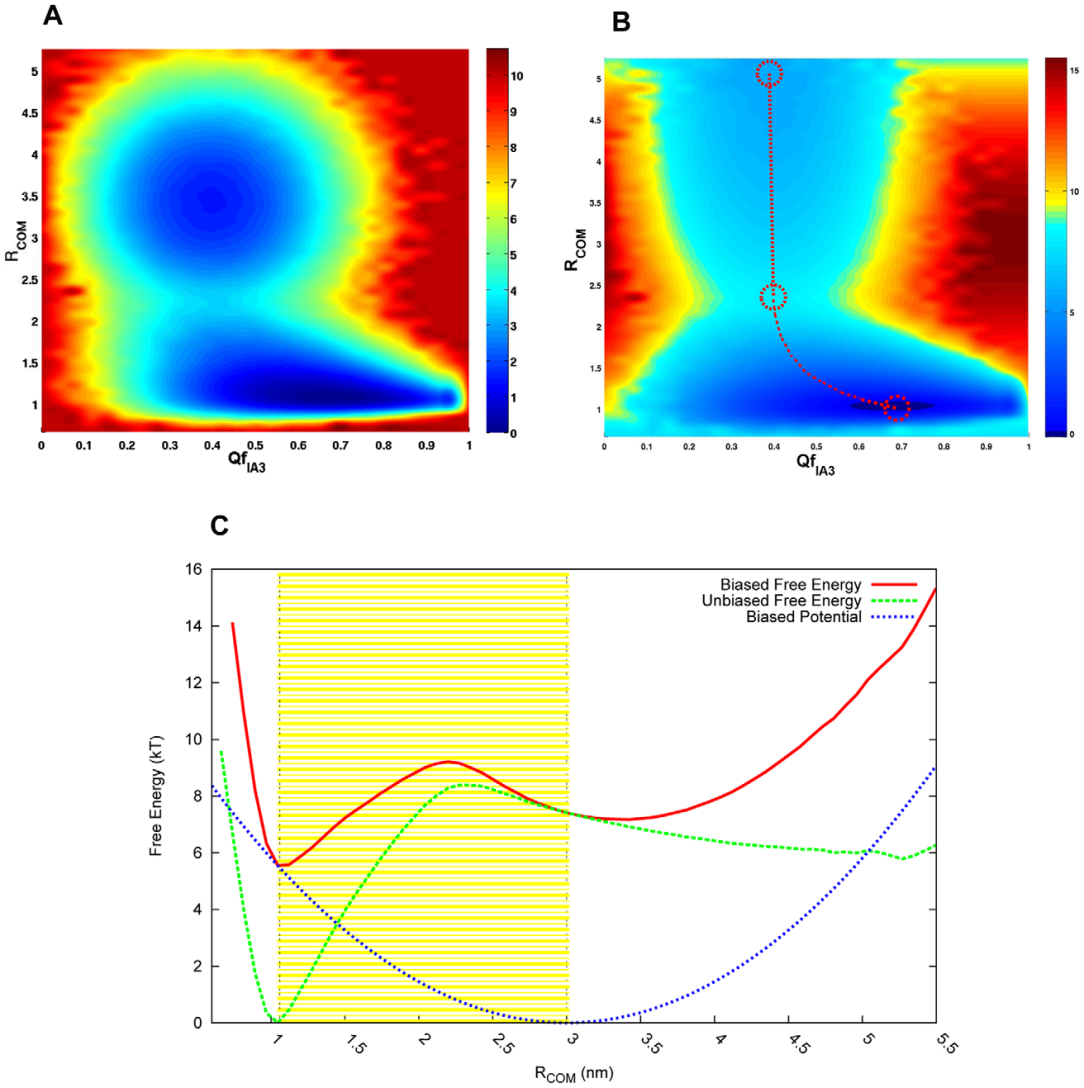
One- and two-dimensional free energy profiles (in units of 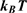) obtained by the coarse grained structure based model at 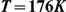. (A) The free energy surface before unbiasing is shown as a function of 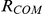 and 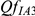. (B) The unbiased free energy surface is shown as a function of 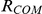 and 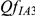. The binding route is marked by the red dotted line. The initial state, transition state and final state are labelled by red dashed circles. The binding route shows that IA3 interacts with YPrA prior to folding into a helical structure. (C) The biased and unbiased free energy profiles and the harmonic biasing potential are shown along the coordinate representing the center of mass distance between YPrA and IA3. The biasing potential is introduced to accelerate the sampling around the important transitions (yellow region), by raising both the native binding state (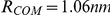) and nonnative unbinding state (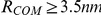) with only a small perturbation at the transition state.

Based on boundary conditions, the initial and final coordinates must be specified. We take the crystalline structure of YPrA complexed with IA3 mutant inhibitor (PDB code: 1DP5) as the endpoint of the transitional trajectories. We assume that the trajectories start with the uninhibited enzyme and unfolded IA3 that is far from the binding site of proteinase A. For free YPrA the coordinates in trigonal and monoclinic crystal forms are accessible from Protein Data Bank under accession codes 1FMU and 1FMX, respectively. Here, we adopt the coordinates of the trigonal crystal form, not only for the clarity of the electron density in the “flap” consisting of a 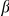 hairpin loop extending over the active site, but also because of the possible presence of some hydrolysis products in the monoclinic crystal [29]. However, in the crystal structure of the trigonal form, there are two disordered regions in which the electron density is relatively poor. The two highly flexible regions, located at the peptide segments 162–165 and 243–245, are considered to make less contribution to the inhibitor binding as their locations on the molecular surface are far from the active site. The missing segments were modelled by structure prediction. Although YPrA is glycosylated its covalently binding carbohydrate moieties are not considered in the simulation. The structural difference between the initial and final states of YPrA is shown in Supplemental Figure 1A in Text S1. Obviously, the unfolded state of the inhibitor can not be represented by a single structure. It should be an ensemble of conformations having a sufficient number of degrees of freedom. These were generated by Langevin dynamics using NAMD [66] with Charmm22 force field. The initial conformation of the system was constructed in stages, starting with the complex of folded IA3 and uninhibited YPrA with a center of mass distance of 3 nm from each other, followed by packing the complex with a 4 nm thick water sphere. We then carried out minimization using the conjugate gradient algorithm with 1000 steps. The initial distance between IA3 and YPrA was set to about 3 nm as hinted by the coarse grained model. The region of most interest is the range 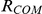 from 1.06 nm to 3.0 nm, where folding and binding occur, which is indicated by the yellow region in Figure 7. In order to generate the conformation of the complex, the minimized system was heated to 500 K in the canonical ensemble. The procedure employed a Langevin thermostat with a 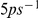 damping parameter. Constraints were applied to the lengths of all bonds involving hydrogen atoms, thus allowing a 2 fs time step. A spherical boundary condition was used to control the 4.5 nm thick water sphere from the center of mass of the complex. YPrA was fixed during the high temperature dynamics. After heating IA3 for 1 ns, we extracted intermediate structures (without water) whose RMSDs from the helical structure were larger than 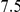 *Å* and that were separated by interval steps larger than 1 ps.

## Supporting Information

Text S1 Supporting information of flexible binding-folding of IA3 to YPrA.
(5.21 MB PDF)
