# Supplementary material for: Multi-Scaled Explorations of Binding-Induced Folding of Intrinsically Disordered Protein Inhibitor IA3 to its Target Enzyme
Source: PLoS Comput Biol. 2011 Apr 7;7(4):e1001118. doi: 10.1371/journal.pcbi.1001118 (PMC3072359; doi:10.1371/journal.pcbi.1001118)
Supplement: Text S1 — Supporting information of flexible binding-folding of IA3 to YPrA. (5.21 MB PDF) [file pcbi.1001118.s001.pdf]

Text S1

1 Microscopic Perspective at Atomic Level

Two hundred paths were calculated and nine were optimized successfully. It is a non-trival task to tune the parameters in the path model for optimizing the stochastic kinetic paths with full-atomic details. The difficuties lie in 1) the side chain-dependent steric clash which may cause high local energy and blow up the system; 2) inreasonable chain crossing during the process due to the protein topological complexity. It is the major reason that we failed to optimize most of the paths. We expect that this will be improved in the future version of Moil. In our calculation, all the nine paths share the similar global mechanism. Their differences lie in the detailed routes and the nucleation position in IA3. In this paper, we choose the three paths (labelled path1, path2, path3 in main text) as concrete examples in an arbitrary way. At first, we count the native contacts in IA3 and interfacial contacts along these paths. It is shown in Fig. S2. We found the formation and breaking of native contacts along the path to the final state. This is particularly clear in path3.

To shed light on the structural evolution from microscopic perspectives, we examine the formation of hydrogen bonds and salt bridges during the process. In Fig. S3 (a), the number of hydrogen bonds of interface (first line), intrachain of IA3 (mid line) and the complex (last line) are plotted along these pathways. Several non-native hydrogen bonds and salt bridges are found both of intrachain of IA3 and interchain between IA3 and YPrA during the folding and binding process. For instance, in $path1$, there are intrachain salt bridges: L7-E10, L16-E17; in $path2$, intrachain salt bridge D4-L7 and interchain hydrogen bond T79A-E17B; in $path3$, interaction network formed by salt bridge L16-E17 and hydrogen bonds Q13-E17 and Q13-L16, interchain hydrogen bond T113A-L18B. They are shown in Fig. S3 (b). It is worthwhile noticing that we didn't find special specific non-native hydrogen bonds and salt bridges on these pathways. The results are consistent with that of coarse grained model.

2 Reaction Coordinate: Q score

It is the total fraction of the target segment of polypeptide to characterize the structural similarity to the referenced structure. Here, the referenced structure is the crystal structure of the enzyme-inhibitor complex (PDB code: 1DP5). Q value is computed by the formula as follow:

$$Q = \frac{2}{(N-1)(N-2)} \sum_{i<j-1} exp\Big[-\frac{(r_{ij}-r_{ij}^N)^2}{2\sigma_{ij}^2}\Big]. \tag{1}$$

Where, N represents the total residue number of target polypeptide, i and j are the residue sequence number. r_{ij} and r_{ij}^N refer to the C_α distances between residue i and j of intermediate conformations and the native complex structure, respectively. σ_{ij} is the width of the function and equal to $|i-j|^{0.15}$ and the normalization $(N-1)(N-2)/2$ is the number of non-nearest neighbor pairs given the total residue number N. The Q value fluctuates between 0 (completely different with non-overlap with the native complex structure) and 1 (perfectly correspond with the final structure).

3 Estimation of Binding Affinity

To estimate the equilibrium disassociation constant K_d reflected binding affinity between IA3 and YPrA, we consider two-state kinetics for the formation of the enzyme-inhibitor complex C from free IA3 and

the protease at equilibrium, which may be expressed as,

$$[I] + [Y] \underset{K_{off}}{\overset{K_{on}}{\rightleftharpoons}} [C] \quad (2)$$

where K_{on} is the second-order rate constant as the association rate, while K_{off} is the first order rate constant for the dissociation. The ratio between the two rate constants yields the equilibrium disassociation constant K_d (has units of concentration) given by:

$$K_d = \frac{K_{off}}{K_{on}} = \frac{[I] * [Y]}{[C]}$$

We define the concentration of free enzyme $[E_f]$ and free inhibitor $[I_f]$, so total enzyme concentrantion $[Y_0] = [Y_f] + [C]$, and total IA3 concentration $[I_0] = [I_f] + [C]$. Given that the effective simulation box is spherial of radius 8.337 nm (the largest R_{COM} in our simulation), the IA3 concentration is inversely proportional to the volume of the sphere, $4\pi R^3/3$. Then $[I_0] = 0.684 * 10^3 \mu M$. Likewise the total enzyme concentration is $[Y_0] = 0.684 * 10^3 \mu M$. ΔG is is about 6 kT at this concentration which is estimated from the free energy difference between free IA3 and the complex state (from figure 7 in main text). Then $\frac{[C]}{[I_f]} = exp^{(\Delta G/KT)}$. So that $K_d = \frac{[I_f][Y_f]}{[C]} = 4.192 nM$.

4 Cut-off Algorithm to Count Native and Non-native Contacts in the Coarse-grained Structure-based Model

We used a cut-off algorithm to describe the interactions between the two chains, which can describe both native and non-native contacts formed in the trajectory. We find that in the native complex the average distance between two C_α atoms that form a contact between the two chains is 8.831 $\AA$. We therefore used two radius cut-offs to define a contact value. If the distance between two C_α atoms is shorter than 6 $\AA$, we define the contact value as 1, while if the distance is between 6 $\AA$ and 10.60 $\AA$ (which is 1.2 times 8.831 $\AA$) the contact value is 0.5. Using this algorithm to describe the native complex, we find that the total inter-chain contact value is 145.5, or a little higher than the 134 that is calculated by SCM.

In order to measure which part of YPrA interacts with IA3 at the transition state, it is easy to use the cut-off algorithm which has been used to count the interfacial contacts in a simple and direct way. However, the cut-off distance should be chosen carefully. If it is too large, non-native contacts are overcounted. If the cut-off is too small, native contacts will be undercounted. In our simplified coarse-grained structure-based model, the non-native contacts are treated as repulsive interactions, and only the native contacts are energetically favorable. Non-native contacts may be overestimated because a distance cutoff algorithm is coarse and cannot avoid finding non-native contacts near a native contact. We separated the non-native contacts into two groups: near-native and away-native. If in a non-native contact pair, both of the C_α atoms are more than 5.0 $\AA$ from any atoms that participate in a native contact pair, this non-native contact is considered as away-native. Otherwise, it is near-native. This is illustrated schematically in Fig. S6, where residues in IA3 and YPrA are represented by magenta and yellow spheres, respectively.

We found that native contacts, near-native non-native contacts, and away-native non-native contacts form 13.4%, 25.8%, and 60.8%, respectively. Therefore, there are more away-native non-native contacts than near-native non-native contacts at the transition state. The non-native contacts are primarily away-native non-native contacts. Overall, the cutoff algorithm can provide useful information about native and non-native contacts in the transition state topology, although it is coarse grained. We have explored more quantitatively the role of non-native interactions of IA3 binding to YPrA by our full-atomic model, using the combination of AMBER and OPLS force field (not the structure-based model).

5 Contact Maps based on SCM

The atomic contact maps are also based on the algorithm of SCM. If there is at least an atom-atom contact between a pair of residues, a contact between this pair of residues is considered to be formed which is represented by a hollow triangle in the plot. In order to describe the degree of strength of interaction between a pair of residues, the corresponding number of atomic contacts existing in the residue pair is shown by the color bar. To make the contacts map more informative, the native contacts are shown by squared points colored according to the number of atomic contacts in the residue pair. In this way, we can estimate that the native contact forms by the triangle with a squared point situated at the center, and the non-native contact forms by a single triangle. Two grey lines are used to divide the regions of intra-chain contacts of IA3 and interfacial contacts. In addition, the contacts of residue pair of which the index separation is less than four are not considered, because in any case these contacts are required to maintain the backbone not being broken. The residue sequence number of aspartic proteinase A is rearranged from 2 to 330, and that of IA3 is from 2 to 32. In the contact map, the residue index of YPrA is from 1 to 329 and that of IA3 is 330 to 360. Noted that, as a concrete example of pathway, only the $path1$ is shown in this supporting information. The contact maps and their corresponding structures on the folding path are shown in Fig. S4 (c). To view the structural evolution clearly, the relation between contact maps and structure is shown in Fig. S4 (a-b). The surface areas in YPrA are labelled A-J. They are colored blue exclude that the two loop regions are red. C region is the "flap". H region is loop2. Along $path1$, the initial and final states are placed at top left corner and left bottom. In the lower right corner, it corresponds to the grid 55. The interfacial contacts are labelled by red rectangles.

It is clear that the interfacial contacts are mostly contributed by non-native interactions before grid 66. The region between grid 55 to 66 may correspond to the transition state region predicted by our coarse grained model. We can also see, after this region, native contacts increase dramatically. From the evolution of contact map, we can also see that IA3 forms partially ordered structure (labelled by blue triangles) only after TS region. Considering together with the formation of native interfacial contacts also after this region, this may support a coupled folding and binding process after transition state consistent with the result by our coarse grained model. In addition, we can see the sequential order of IA3 binding to YPrA. It seems that the first regions which IA3 contacts are loop1 (labelled by C) and partial D region. The second region is loop2 (labelled by H). Then it is the region J. The four regions (C,D,H,J) all locate on the outer surface of the active site groove. After IA3 entering the groove, it contacts the surface at the bottom of the groove (labelled by A,B,E,F,G,I). In addition, we found that several non-native hydrogen bonds and salt bridges formed during the process. For example, intrachain salt bridges: L7-E10, L16-E17 and interchain hydrogen bonds: T79A-E17B, T246A-Q5B. It is worthwhile pointing out that, among 8 pathways we calculated, in four paths of them there is a nonnative intrachain salt bridge: L16-E17. This special nonnative salt bridge can neutralize partial charges in acidic and basic sidechains and contribute the stabilization of IA3. The reason of this salt bridge forming more frequently may be that sidechains of the two neighboring residues are able to contact easily in topology. we also found that hydrophobic cluster2 forms at first at grid 57. It contributes most part of non-native contacts at the first stage that IA3 bind to the outer surface of YPrA (C,D regions). Note that, this case may not be inevitable along other paths in the multi-dimensional energy landscape.

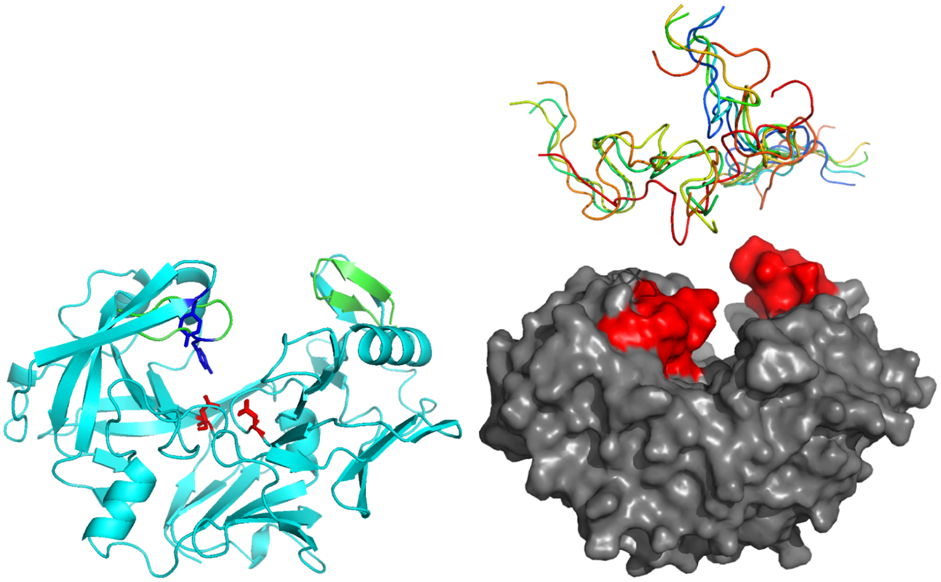

(a) (b)

Figure 1. (a) Structural alignment between the initial state and final state of YPrA during this simulation. Their conformations are modelled based on the structures of proteinase A alone in trigonal form (1FMU) and its inhibited form by N terminal IA3 mutant (1DP5). Superimposition of the conformation of initial point onto that of the final point reveals an overall RMSD of 0.59 $\AA$. The structural distinction is reflected in two loops located on the surface of the two domains. One is the flap and the other one is the segment 243-249 in the C-terminal domain whose coordinates can not be captured by X-ray crystal diffraction in trigonal crystal of free PrA. The two loops in free form are colored in cyan to emphasize their structural diversity from the complex form. The other parts of the proteinase are quite stable upon inhibitor binding including the two catalytic aspartic acid residues even more immobile which are colored red. (b) The initial structures of all the pathways we have calculated. The different initial structures of IA3 are represented by coils with different colors. The two loop regions are colored in red.

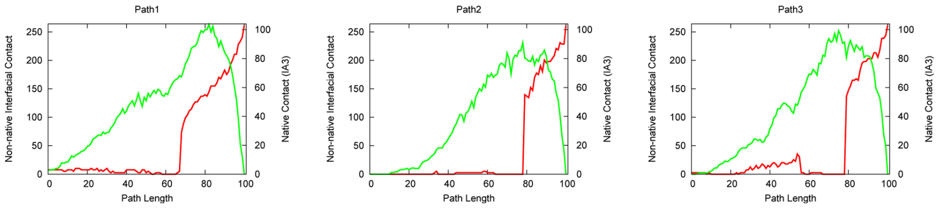

(a) (b) (c)

Figure 2. The non-native interfacial contact curves (green line) overlaid with native contact curve of N terminal of IA3 (red line) along the folding pathway of $path1$ (a), $path2$ (b) and $path3$ (c). We found the formation and breaking of native contacts along the path to the final state. This is particularly clear in $path3$.

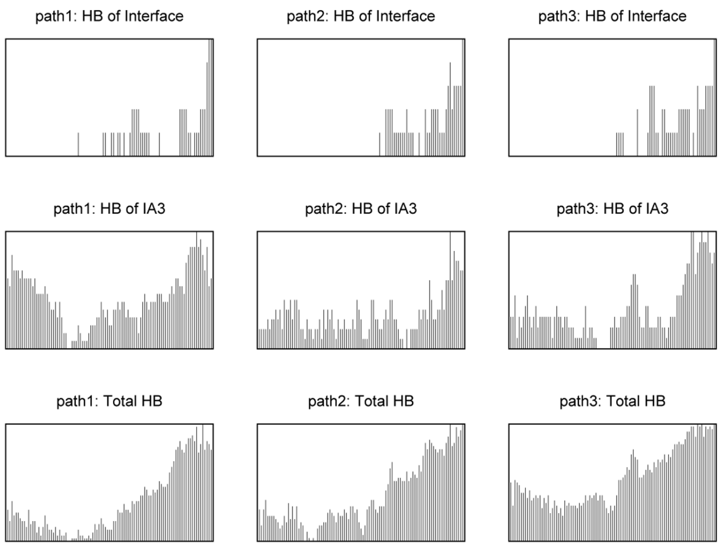

(a)

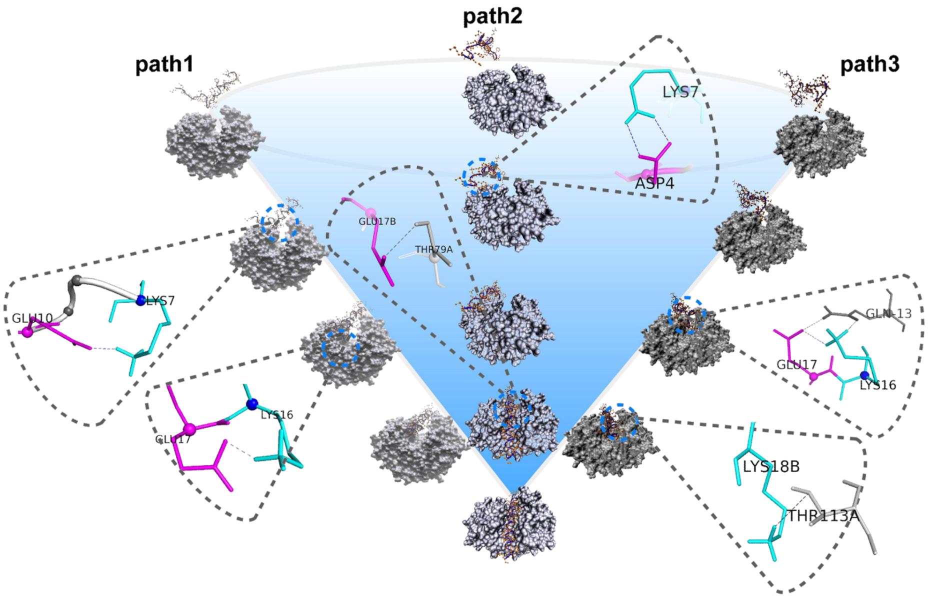

(b)

Figure 3. (a) Evolution of number of hydrogen bonds on *path*1, *paht*2 and *path*3. In first row, it is the hydrogen bonds formed in interface. Hydrogen bonds formed in N terminal of IA3 and the complex are shown in second row and third row. The three columns correspond to *path*1, *paht*2 and *path*3, respectively. (b) Schematic structural evolution along these three paths. On the route, we found several non-native hydrogen bonds and salt bridges formed during the process. They are emphasized and shown at the side of structural graphs. The hydrogen bonds and salt bridges are illustrated in dashed lines. Basic residues are colored in cyan sticks and blue spheres and acidic residues are represented by magenta sticks and spheres. For sake of clarity, we only show part of typical non-native hydrogen bonds and salt bridges. On *path*1, there are intrachain salt bridges: L7-E10, L16-E17; on *path*2, intrachain salt bridge D4-L7 and interchain hydrogen bond T79A-E17B; on *path*3, interaction network formed by salt bridge L16-E17 and hydrogen bonds Q13-E17 and Q13-L16, interchain hydrogen bond T113A-L18B.

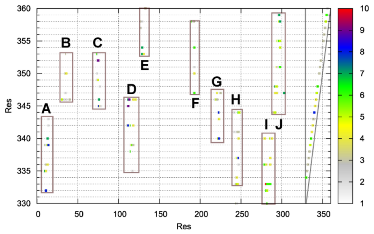

(a)

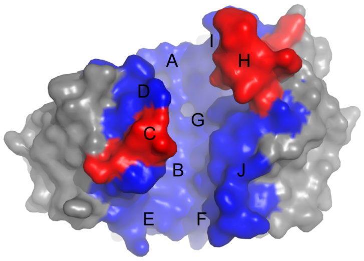

(b)

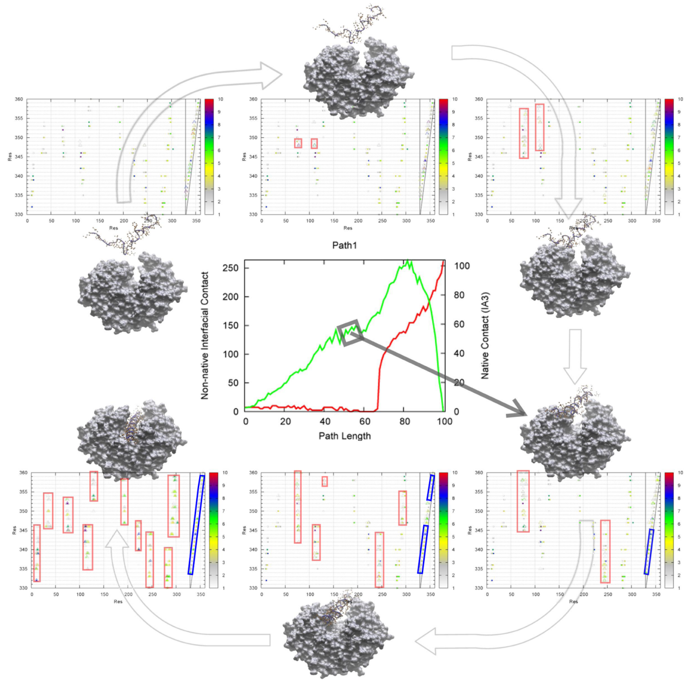

(c)

Figure 4. Native contact map of interface between N terminal of IA3 and YPrA and its corresponding regions in YPrA and the contact map evolution along the pathway: $path1$**.** (a) Native contact map in which the interfacial contacts are located on the surface of active site groove in YPrA. (b) Corresponding regions labelled A to J according to the interfacial contact map. They are colored blue exclude that the two loop regions are red. C region is the "flap". H region is loop2. (c) Contact map evolution along $path1$ and represented structures.

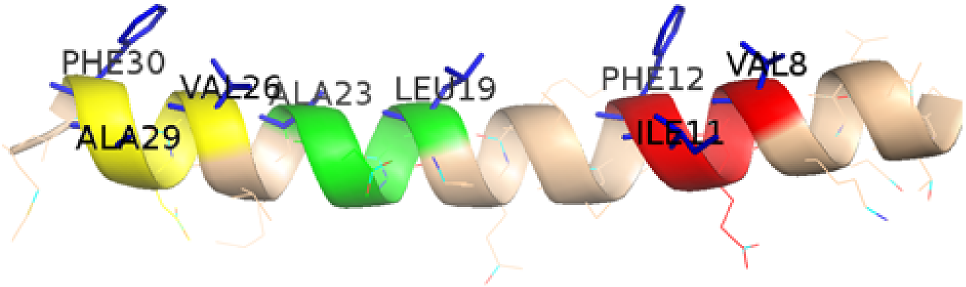

Figure 5. The helix structure of N terminal IA3 (residues 1-31) in cartoon. In the crystal structure of the complex, the hydrophilic face of IA3 is oriented toward the solvent, the other face enveloped completely with the residues of the active site cleft, is composed of nine hydrophobic amino acid residues, V8, I11, F12, L19, A23, V25, V26, A29 and F30. They constitute three hydrophobic clusters, "cluster-1" (red) of V8-X-X-I11-F12 in the N-terminal, "cluster-2"(green) of L19-X-X-X-A23 in the mid, and the C-terminal "cluster-3"(yellow) of V26-X-X-A29-F20.

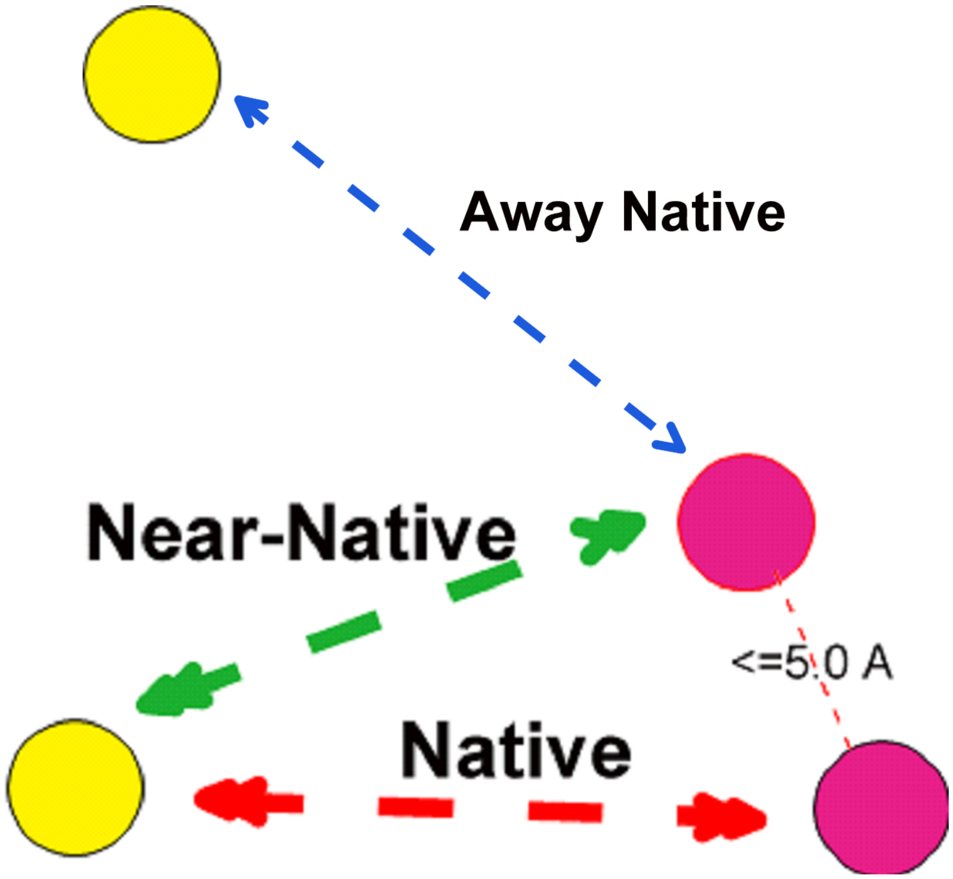

Figure 6. Schematic diagram of contacts calculation by cut-off algorithm. We used two radii to define the contact. If the distance of two C_α atoms is shorter than 6 $\mathring{A}$, we set the contact value 1, while the distance is between 6 $\mathring{A}$ and 10.60 $\mathring{A}$, which is 1.2 times average distance of two C_α atoms at native state, the contact value is 0.5. We separated the non-native contacts into two parts: near-native and away-native non-native contacts. If a non-native contact pair whose Ca atoms are not both within 5.0 Angstrom of any atoms in a native contact pair, this non-native contact is considered as away-native. Otherwise, it is near-native. Residues in IA3 and YPrA are represented by magenta and yellow spheres, respectively.
